# The risks and benefits of patients temporarily discontinuing medications in the event of an intercurrent illness: a systematic review protocol

**DOI:** 10.1186/s13643-015-0135-y

**Published:** 2015-10-24

**Authors:** Andrew Morden, Jeremy Horwood, Penny Whiting, Jelena Savovic, Laurie Tomlinson, Thomas Blakeman, Charles Tomson, Alison Richards, Tracey Stone, Fergus Caskey

**Affiliations:** NIHR CLAHRC West, Bristol, UK; School of Social and Community Medicine, University of Bristol, Bristol, UK; Department of Non-communicable Disease Epidemiology, London School of Hygiene and Tropical Medicine, London, UK; Centre for Primary Care, Institute of Population Health, The University of Manchester, Manchester, UK; NIHR CLAHRC Greater Manchester, Manchester, UK; Department of Renal Medicine, Freeman Hospital, Newcastle Upon Tyne Hospitals Foundation Trust, Tyne and Wear, UK; UK Renal Registry, Bristol, UK

**Keywords:** Acute kidney injury, Medication discontinuation, Sick day rules, Diuretics, Angiotensin-converting enzyme inhibitors, Angiotensin receptor blockers, Direct renin inhibitors, NSAID, Metformin and sulfonylureas

## Abstract

**Background:**

Acute kidney injury (AKI) is common and often leads to significant morbidity and/or death. The development of AKI, or complications associated with it, may be due to use of certain medications in at-risk patients experiencing an intercurrent illness. Implicated drugs include diuretics, angiotensin-converting enzyme inhibitors/angiotensin receptor blockers/direct renin inhibitors, non-steroidal anti-inflammatory drugs (NSAIDs), metformin and sulfonylureas. Expert consensus opinion (and clinical guidelines) recommend considering discontinuation of diuretics, angiotensin-converting enzyme inhibitors/angiotensin receptor blockers/direct renin inhibitors, NSAIDs, metformin and sulfonylureas in the event of an intercurrent illness to prevent AKI onset or reduce severity or complications. However, the evidence base for these recommendations is very limited. This systematic review aims to address the available evidence for the temporary discontinuation of diuretics, ACE inhibitors, angiotensin receptor blockers, direct renin inhibitors, non-steroidal anti-inflammatories and metformin and sulfonylureas for those at risk of AKI or with newly diagnosed AKI.

**Methods/Design:**

Randomised controlled trials; non-randomised trials; cohort studies; case-control studies; interrupted time series studies; and before-and-after studies featuring adults aged 18 and over in any setting currently taking diuretics, angiotensin-converting enzyme inhibitors/angiotensin receptor blockers/direct renin inhibitors, NSAIDs and metformin; experiencing an intercurrent illness; or undergoing a radiological/surgical procedure (planned or unplanned) will be searched for. Relevant trial registers and systematic review databases will be searched. Systematic reviews will be assessed for methodological quality using the ROBIS tool, trials will be assessed using the Cochrane risk of bias tool, and observational studies will be assessed using the ACROBAT-NRS tool. If sufficient studies assessing similar populations, study type, settings and outcomes are found, then a formal meta-analysis will be performed to estimate summary measures of effect. If not, a narrative synthesis will be adopted.

**Discussion:**

This review will synthesise evidence for the efficacy of discontinuing diuretics, angiotensin-converting enzyme inhibitors/angiotensin receptor blockers/direct renin inhibitors, NSAIDs, metformin or sulfonylureas to prevent or delay onset of AKI or associated complications. Results will provide guidance on efficacy and safety of this strategy and potentially help to develop an intervention to test the best mechanism of guiding medication discontinuation in at-risk populations.

**Systematic review registration:**

PROSPERO CRD42015023210

**Electronic supplementary material:**

The online version of this article (doi:10.1186/s13643-015-0135-y) contains supplementary material, which is available to authorized users.

## Background

Acute kidney injury (AKI) is a comparatively recent term describing a sudden reduction in renal excretory function. The term was introduced because of a lack of agreement about what compromised ‘acute renal failure’, with some definitions only including the requirement for renal replacement therapy. The causes of AKI can be divided into three groups: pre-renal, such as low blood pressure; intrinsic renal, commonly the result of prolonged sepsis or hypotension but also resulting from immune conditions affecting the kidney; and post-renal, caused by partial or complete obstruction to the flow of urine from the kidneys [1]. There are a number of key risk factors for AKI, including the following: existing chronic kidney disease (CKD), aged >65 years, cardiac failure, liver disease, diabetes mellitus, use of nephrotoxic drugs such as non-steroidal anti-inflammatories (NSAIDs) and aminoglycosides, hypotension (compared to baseline blood pressure) and sepsis, neurological or cognitive impairment or disability, or prior urological obstruction [1, 2]. An episode of AKI may be triggered by an intercurrent illness, such as diarrhoea and vomiting, influenza, other viruses, urinary tract infections, respiratory tract infections, skin infections and hypovolaemia from any cause in those most at risk [1, 3].

The pooled incidence rate of AKI globally is 21.6 % in adults [4], and AKI is predicted to feature in 1–7 % of hospital admissions [1, 3, 5–7]. The morbidity and mortality rates associated with an episode of AKI (in hospital and after discharge) are high [6, 8–10]. Coca et al.’s systematic review reports that the incidence of CKD and end-stage kidney disease (ESKD) after an AKI episode is 7.8 and 4.9/100 patient-years, respectively, and that AKI is associated with long-term mortality [10].

Pannu and colleagues review the potentially harmful biochemical effects of medications that are administered as part of the routine care of critical illnesses in hospital settings and conclude that many, such as angiotensin-converting enzyme (ACE) inhibitors and NSAIDS, could be detrimental to kidney function [9]. Medication-induced episodes of kidney problems are increasingly prevalent and indeed doubled between 1999 and 2009 in the UK [11]. Commonly prescribed medications including diuretics, ACE inhibitors/angiotensin receptor blockers/direct renin inhibitors, NSAIDs, metformin and sulfonylureas have been associated with AKI (and related complications) in a variety of settings [12–30]. The incidence of AKI is higher amongst patients who have multiple comorbidities and who are taking combinations of drugs which may interact to enhance the physiological insult underlying AKI [31].

Expert consensus statements and clinical guidelines have recommended that clinicians temporarily withhold potentially harmful medications for high-risk patients when they develop an intercurrent illness to reduce risk of AKI [1–3]. Other interventions have gone further by suggesting that patients stop the medications when they become unwell [32]. However, there is surprisingly little evidence to support these recommendations. The National Institute for Health and Clinical Excellence (NICE) guidelines issued in 2013 found no studies which evaluate the efficacy of medication discontinuation during an intercurrent illness in terms of preventing onset or harm from AKI [2]. There is growing concern regarding the overall risks and benefits of the sick day rules approach [33]. Such advice could lead to an increase in complications from the conditions which the drugs are prescribed such as heart failure and hypertension. NSAIDs are recommended for pain control, especially for osteoarthritis pain in older adults [34]; cessation of NSAIDs may result in a marked reduction in mobility and make the difference between a person coping at home or not. In addition, it is suggested that continuing some of these drugs will have renal and cardiac benefits, even in the presence of AKI, at least in some patients. ACE inhibitors and angiotensin receptor blockers/direct renin inhibitors, for instance, preserve tubular blood flow and may prevent the tubular injury that characterises the transition from reversible pre-renal AKI to established, intrinsic renal AKI, which has a poorer prognosis [35].

Therefore, the overall objective of this review is to assess the available evidence for the temporary discontinuation of diuretics, ACE inhibitors, angiotensin receptor blockers, direct renin inhibitors, non-steroidal anti-inflammatories, metformin and sulfonylureas in adults in primary and secondary care at risk of AKI or with newly diagnosed AKI as a result of an intercurrent illness or a radiological/surgical procedure (planned or unplanned). To address this objective, we will answer the following review questions:

1. What are the effects of temporary discontinuation of diuretics, angiotensin-converting enzyme inhibitors/angiotensin receptor blockers/direct renin inhibitors, NSAIDs, metformin and sulfonylureas in those at risk of AKI?
2. What are the effects of the temporary discontinuation of diuretics, angiotensin-converting enzyme inhibitors/angiotensin receptor blockers/direct renin inhibitors, NSAIDs, metformin and sulfonylureas for the progression of AKI across AKI severity categories?
3. What are the adverse effects associated with stopping diuretics, angiotensin-converting enzyme inhibitors/angiotensin receptor blockers/direct renin inhibitors, NSAIDs, metformin and sulfonylureas?
4. What is the effectiveness of interventions to encourage stopping of diuretics, angiotensin-converting enzyme inhibitors/angiotensin receptor blockers/direct renin inhibitors, NSAIDs, metformin and sulfonylureas?

## Methods/Design

Systematic review methods will follow the principles outlined in the Centre for Reviews and Dissemination (CRD) guidance for undertaking reviews in health care [36] and the Cochrane Handbook [37]. The protocol is registered on the PROSPERO database (CRD42015023210). The review will be reported according to the PRISMA guidelines [38, 39]. This protocol follows the PRISMA-P reporting guidelines [40].

### Eligibility criteria

Studies that fulfil the following criteria will be eligible for inclusion:

### Types of studies

The following types of studies will be included: randomised controlled trials, non-randomised trials, cohort studies, case-control studies, interrupted time series studies, before-and-after studies. If high-quality systematic reviews are identified that fulfil all inclusion criteria for the review, then these will be eligible for inclusion. We will include the systematic review if it includes all relevant studies for a single research question; if additional studies fulfil our inclusion criteria, then the original review will be used only as a source of relevant studies.

### Participants

Only studies conducted in humans will be sought. Eligible participants will be adults aged 18 and over in any setting (e.g. community, GP surgery, hospital) currently taking diuretics, ACE inhibitors, angiotensin receptor blockers, direct renin inhibitors, non-steroidal anti-inflammatories, metformin or sulfonylureas; experiencing an intercurrent illness; or undergoing a radiological/surgical procedure (planned or unplanned).

### Interventions

Temporary discontinuation of diuretics, angiotensin-converting-enzyme inhibitors/ angiotensin receptor blockers / direct renin inhibitors, NSAIDs, metformin and sulfonylureas as a result of a specific intervention or for any other reason. Eligible comparator interventions (if present) will be placebo, no treatment or usual care.

### Outcomes

The review will be restricted to studies that report some measure of kidney function. We anticipate that other relevant outcomes will include all-cause mortality, cardiac events, diabetic events and pain-related outcomes (pain severity, use of GP services or admission to hospital relating to NSAID withdrawal).

### Search strategy

Attempts will be made to identify relevant studies on temporarily discontinuing diuretics, angiotensin-converting enzyme inhibitors/angiotensin receptor blockers/direct renin inhibitors, NSAIDs, metformin and sulfonylureas. Search methods will meet best practice standards in systematic reviews [36, 37]. The search strategies will be developed specifically for each database and the keywords adapted according to the configuration of each database. Searches will not be limited by language, date or publication status (unpublished or published). Searches will be performed in multiple databases from inception to present, including Embase (OvidSP), Medline (OvidSP), Medline In-Process Citations & Daily Update (OvidSP), PsycINFO (OvidSP), BIOSIS Citation Index (Web of Science), CINAHL (Cumulative Index to Nursing and Allied Health Literature) (EBSCO), Science Citation Index (SCI) (Web of Science) and Cochrane Central Register of Controlled Trials (CENTRAL) (Wiley).

Supplementary searches will be undertaken to identify grey literature, completed and ongoing trials, in resources such as NIH ClinicalTrials.gov (http://www.clinicaltrials.gov), metaRegister of Controlled Trials (http://www.controlled-trials.com) and WHO International Clinical Trials Registry Platform (ICTRP) (http://www.who.int/ictrp/en); relevant guidelines (i.e. NICE in the UK) regarding management of AKI; and references of published articles found in the search***.*** An example of the search strategy is presented in Additional file 1.

Identified references will be downloaded into EndNote X7 software for further assessment and handling. Rigorous records are maintained as part of the searching process. Individual records within the EndNote reference libraries will be tagged with search information, such as searcher, date searched, database host, database searched, strategy name and iteration, theme, or search question. This will enable the information specialist to track the origin of each individual database record and its progress through the screening and review process. A review-specific access database will be used to manage screening and data extraction.

### Selection of studies

Two reviewers will independently screen the titles and abstracts of all reports identified by searches, and any discrepancies will be discussed and resolved by consensus. Full copies of all studies deemed potentially relevant will be obtained, and the same two reviewers will independently assess these for inclusion; any disagreements will be resolved by consensus.

### Data extraction

Data extraction will be carried out using standard data extraction forms designed specifically for this review. Data will be extracted by one reviewer, using a piloted, standard data extraction form, and checked by a second reviewer; any disagreements will be resolved by consensus. Data will be extracted on the following: participant characteristics, study design, inclusion and exclusion criteria, details of intervention (if applicable—potentially including an outline of the characteristics of the interventions in terms of the (1) format and content of any ‘sick day rules’ advice and (2) whether part of a wider package of care, i.e. in the context of other AKI/kidney health initiatives), details of outcomes assessed (primary and other outcome measures) and results. If, during the course of the review, outcome measures commonly reported in studies are found these will be included and documented [40].

#### Quality assessment

Systematic reviews will be assessed for risk of bias using the ROBIS tool [41]: this tool’s aims include domains covering study eligibility criteria, identification and selection of studies, data collection and study appraisal, synthesis and findings, and interpretation. Trials will be assessed for methodological quality using the Cochrane risk of bias tool [37]. This includes items covering selection bias (random sequence generation and allocation concealment), performance bias (participant blinding), detection bias (blinding of outcome assessors), attrition bias (incomplete outcome data) and reporting bias (selective reporting). There is also an additional field for other sources of bias. We believe that all important concerns about bias are included in the other domains in the tool and so no further domains will be added. We will use the new ACROBAT-NRS tool to assess the risk of bias in observational studies [42]. It includes domains covering bias due to confounding, bias in the selection of participants into the study, bias due to departures from intended interventions, bias due to missing data, bias in taking measurements and bias in the selection of the reported result. For all tools, if at least one of the domains is rated as ‘high’, the study will be considered at high risk of bias; if all domains are judged as ‘low’, the trial will be considered at low risk of bias; otherwise, the trial will be considered at ‘unclear’ risk of bias. The risk of bias assessment will be conducted as part of the data extraction process.

### Data synthesis

A narrative summary of the included studies will be presented. This will include a summary of the characteristics (e.g. study design, intervention (if applicable), population size, geographical location, settings (community, hospital or other healthcare settings), year, baseline population characteristics and outcome definition). If data are considered too heterogeneous to pool, or not reported in a format suitable for pooling (e.g. data reported as medians), then we will employ a narrative synthesis. This will involve the use of descriptive text and tables to summarise data in order to allow the reader to consider outcomes in the light of differences in study designs and potential sources of bias for each of the studies being reviewed. This involves organising the studies by (as appropriate) population, outcomes assessed, type of study and study settings; summarising the results of the studies, summarising the range and size of the associations these studies report; and describing the most important characteristics of the included studies. A detailed commentary on the major methodological problems or biases that affected the studies will also be included, together with a description of how this has affected the individual study results.

If sufficient studies assessing similar populations, study type, settings and outcomes are found, then a formal meta-analysis will be used to estimate summary measures of effect. For dichotomous data, we will calculate the relative risk (RR) for each study with the associated 95 % confidence intervals. Continuous data will be analysed as the mean difference between groups and associated 95 % confidence intervals. For multi-arm studies, we will analyse each intervention arm compared to the control group separately.

We anticipate that systematic differences between studies (heterogeneity) will be likely. Therefore, the random effects model will be used to calculate summary estimates. Heterogeneity will be investigated visually using forest plots and statistically using the *I*^2^ and *Q* statistics [43].

Heterogeneity will be formally investigated using meta-regression analyses, where sufficient data are available. We anticipate that the effects of the following variables will be investigated: methodological quality of the primary studies, how the advice or decision to discontinue medications was administered and length of medication discontinuation, age, sex, and comorbidities. Other variables considered relevant on further examination of the literature or input from clinical experts may also be considered. The coefficient describing the effect of each variable on the outcome will be modelled, using a random effects model.

If appropriate, small-study effects (publication bias) will be assessed using a modified linear regression test for funnel plot asymmetry as recommended where there are sufficient numbers of trials (i.e. six trials) [44].

#### Confidence in cumulative evidence

We will consider using the GRADE guidelines [45] to help us evaluate the cumulative quality of the evidence reported in this review for risk of bias, publication bias, imprecision, inconsistency, indirectness and magnitude of effect.

## Discussion

Diuretics, angiotensin-converting enzyme inhibitors, angiotensin receptor blockers, direct renin inhibitors, NSAIDs, metformin and sulfonylureas in at-risk patients experiencing an intercurrent illness are extremely commonly prescribed, and expert consensus (subsequently incorporated into clinical guidelines) recommends the discontinuation of these medications for individuals at risk of AKI during an intercurrent illness. The benefit from such advice is however unproven with scant evidence regarding the benefits and potential harms that discontinuation may confer. An up-to-date systematic review of the evidence on discontinuing medications is warranted. This review will provide evidence on the effectiveness and safety of discontinuing diuretics, angiotensin-converting enzyme inhibitors/angiotensin receptor blockers/direct renin inhibitors, NSAIDs, metformin and sulfonylureas in relation to AKI. We anticipate that the findings will aid healthcare practitioners and policymakers in developing recommendations regarding advice on temporary drug cessation in patients at risk of AKI. Furthermore, the findings will benefit researchers by highlighting future research directions through the identification of gaps in knowledge and shortcomings of the existing evidence base.

## Additional file

Additional file 1:
**Search strategy Medline.** (DOC 68 kb)

## Abbreviations

ACE: angiotensin-converting enzyme
AKI: acute kidney injury
CKD: chronic kidney disease
CRD: Centre for Reviews and Dissemination
ESKD: end-stage kidney disease
NICE: National Institute for Health and Clinical Excellence
NSAIDS: non-steroidal anti-inflammatory drugs
